# Whole-Genome Sequence of a Bordetella pertussis Brazilian Vaccine Strain

**DOI:** 10.1128/genomeA.01570-14

**Published:** 2015-02-19

**Authors:** M. A. Akamatsu, M. Y. Nishiyama, M. Morone, U. C. Oliveira, M. F. B. Bezerra, M. A. Sakauchi, I. Raw, I. L. M. Junqueira de Azevedo, J. P. Kitajima, E. Carvalho, P. L. Ho

**Affiliations:** a Divisão de Desenvolvimento Tecnológico e Produção, Instituto Butantan, São Paulo, Brazil; b Laboratório Especial de Toxinologia Aplicada, Centro de Toxinas, Resposta Imune e Sinalização Celular (CeTICS), Instituto Butantan, São Paulo, Brazil; c Centro de Biotecnologia, Instituto Butantan, São Paulo, Brazil; d Mendelics Análise Genômica, São Paulo, Brazil

## Abstract

Despite the reduction in incidence after vaccination, pertussis disease is still considered a public health problem worldwide, mainly due to recent and potential new outbreaks. We report here the complete genome of the Bordetella pertussis Butantan strain used in the Brazilian National Immunization Program as a whole-cell pertussis antigen to compose vaccines such as DTwP (diphtheria, tetanus, and whole-cell pertussis).

## GENOME ANNOUNCEMENT

Pertussis or “whooping cough” is a contagious respiratory disease caused by Bordetella pertussis. Two vaccines are available, whole-cell pertussis (wP) vaccine and acellular pertussis (aP) vaccine. Recent changes in the epidemiology of pertussis strongly suggest a diminished duration of protection afforded by childhood aP, compared with that of wP vaccine. The additional antigenic components in wP vaccines might induce immune responses with greater durability (1–6).

The introduction of vaccination has reduced the incidence of whopping cough. However, the risk of outbreaks is still a public health concern since several countries have reported an increased incidence of pertussis (2, 7–10). A possible explanation for this is the expansion of strains antigenically distinct from those in the vaccines or a higher waning of immunity among people vaccinated with aP than among those vaccinated with wP. The Brazilian National Immunization Program uses the wP vaccines produced at the Instituto Butantan. In this work, the complete genome of B. pertussis strain 137 (Butantan strain or Bp137), used for the production of the wP vaccine, is described.

Whole-genome sequencing of B. pertussis Butantan strain was performed combining shotgun and paired-end pyrosequencing in 454 GS JR, paired-end sequencing in MiSeq-Illumina, shotgun sequencing in Ion Torrent-PGM and Single Molecules Real-Time sequencing in a PacBio RSII system, leading to a 218-fold coverage of the B. pertussis genome. A preassembly with PacBio reads was performed using HGAp, and the final assembly was done with CLC Genomics Workbench version 6.4 using all reads. The whole genome consists of 4,134,593 bp, with a GC content of 67.7%.

Annotation was obtained by homology, based on sequence similarity to the Tohama I genome, while sequences without homology to Tohama I genes were submitted for prediction by GenMark and compared to sequences deposited in GenBank and the Uniprot Swiss-Prot protein databases. There are 3,462 protein-coding sequences, as well as 51 tRNA and 9 rRNA genes in the genome. A total of 223 copies of insertion element IS481 were identified.

Compared with the Tohama I genome, there were 43 positional changes, including 22 inversions. These regions were mainly flanked by insertion elements; indeed, insertion elements are considered important to promote rearrangement changes in bacterial genomes, especially for Bordetella species (11, 12).

A total of 1,254 single-nucleotide polymorphisms (SNPs) between B. pertussis strain 137 and Tohama I, consisting of 462 missense variations, 273 silent mutations, 280 indels, and 239 noncoding mutations in intergenic regions.

Multilocus sequence type (MLST) analysis showed that B. pertussis strain 137 belongs to clonal complex II. Moreover, the virulence factors were also identified as being PrnA(7), PtxA(4), PtxC(1), *ptxP*(2), Fim2(2), Fim3(1), TcfA(2), OmpQ(2), and Vag8(2) alleles. Many authors associate the pertussis resurgence with genotypic variation in some virulence factors or their promoters (13–16). Interestingly, Prn(1), PtxA(2), *ptxP*(1), and Fim2(1) alleles have been described for several vaccine strains, distinct from the Butantan strain successfully used in massive immunization program in Brazil since the 1980s (13, 14).

The complete genome sequence of a vaccinal B. pertussis strain is an important development that may help in understanding the immune response against B. pertussis vaccination and may also contribute to an explanation of the immune evasion mechanisms of clinical B. pertussis isolates.

### Nucleotide sequence accession number.

The whole-genome sequence of B. pertussis strain 137 has been deposited in GenBank under the accession number CP010323.
